# Changes in Vascular, Lymphatic, Inflammatory, and Lipid Mediators During a 7-Month Calorie-Restricted Low-Carbohydrate, High-Fat Dietary Intervention in Women with Lipedema: A Preliminary Prospective Study

**DOI:** 10.3390/nu18091381

**Published:** 2026-04-28

**Authors:** Angelika Chachaj, Mariusz Fleszar, Łukasz Lewandowski, Paulina Fortuna, Gabriela Maciejewska, Monika Sowicz, Agnieszka Adaszyńska, Urszula Jakobsche-Policht, Małgorzata Krzystek-Korpacka, Andrzej Szuba, Małgorzata Jeziorek

**Affiliations:** 1 Department of Angiology and Internal Medicine, Wroclaw Medical University, 50-367 Wroclaw, Poland; monika.sowicz@wp.pl (M.S.); agnieszkarogala87@gmail.com (A.A.); urszula.jakobsche-policht@umw.edu.pl (U.J.-P.); andrzej.szuba@umw.edu.pl (A.S.); 2 Omics Research Centre, Wroclaw Medical University, 50-368 Wroclaw, Poland; mariusz.fleszar@umw.edu.pl (M.F.); paulina.fortuna@umw.edu.pl (P.F.); gabriela.maciejewska@umw.edu.pl (G.M.); 3 Department of Biochemistry and Immunochemistry, Wroclaw Medical University, 50-368 Wroclaw, Poland; lukasz.lewandowski@umw.edu.pl (Ł.L.); malgorzata.krzystek-korpacka@umw.edu.pl (M.K.-K.); 4 Department of Dietetics and Bromatology, Faculty of Pharmacy, Wroclaw Medical University, 50-367 Wroclaw, Poland; malgorzata.jeziorek@umw.edu.pl

**Keywords:** lipedema, low-carbohydrate, high-fat diet, angiogenic mediators, lymphatic function, endocannabinoid-related lipids, adipose tissue pain, leg volume

## Abstract

**Background/Objectives:** Lipedema is a chronic adipose tissue disorder characterized by disproportionate fat accumulation, pain, microvascular dysfunction, and low-grade inflammation. Although low-carbohydrate, high-fat (LCHF) dietary approaches are increasingly used in clinical practice, their longer-term associations with vascular, lymphatic, and immunometabolic pathways in lipedema remain insufficiently understood. This preliminary exploratory study evaluated clinical outcomes and circulating mediators during a 7-month LCHF dietary intervention. **Methods:** Twenty-four women with lipedema (median age: 39 years) underwent a 7-month individualized, calorie-restricted LCHF diet under medical supervision. Outcomes included body mass index (BMI), leg volume, and adipose tissue pain assessed using a visual analogue scale (VAS). Fasting serum samples collected at baseline and follow-up were analyzed for angiogenic, inflammatory, endothelial, and lipid mediators using Luminex assays and liquid chromatography–tandem mass spectrometry (LC-MS/MS). **Results:** The intervention was associated with significant reductions in BMI, leg volume, and adipose tissue pain (*p* < 0.001). These changes were accompanied by increased vascular endothelial growth factor A (VEGF-A), vascular endothelial growth factor D (VEGF-D), and angiopoietin-2 (Ang-2), together with decreased pro-inflammatory cytokines and endothelial adhesion molecules. Several endocannabinoid-related lipid mediators, including oleoyl ethanolamide (OEA), arachidonoyl ethanolamide (AEA), and palmitoyl ethanolamide (PEA), also decreased. Baseline OEA and AEA concentrations, as well as reductions in OEA over time, were associated with greater BMI reduction. Change in interleukin-8 (IL-8) showed a nominal association with leg volume reduction, while pain improvement was associated with decreases in P-selectin and VEGF-A and increases in interleukin-13 (IL-13). **Conclusions:** A 7-month calorie-restricted LCHF dietary intervention in women with lipedema was associated with clinical improvement and changes in circulating vascular, inflammatory, and lipid mediators. These findings reflect systemic changes accompanying the intervention; however, causal relationships and specific mechanisms cannot be established.

## 1. Introduction

Lipedema is a chronic disorder of adipose tissue, characterized by the bilateral and symmetrical accumulation of subcutaneous fat, most commonly affecting the lower extremities and, less frequently, the arms. Disproportionate subcutaneous fat accumulation is accompanied by adipose tissue pain, tenderness, and easy bruising [1,2]. Clinically, lipedema is diagnosed based on characteristic features, including symmetrical fat distribution, pain in the affected tissue, an increased bruising tendency, and a negative Stemmer’s sign. It is further classified into five types and four stages reflecting fat distribution and disease progression [2].

Lipedema predominantly affects women and is often resistant to conventional weight-loss strategies, including calorie-restricted diets or physical activity [2].

Lipedema is estimated to affect up to 10% of women worldwide. However, the true prevalence remains uncertain due to underdiagnosis and the lack of standardized diagnostic criteria. Comparable estimates have been reported in Europe, while country-specific data, including those from Poland, remain limited [2].

Although the etiology of lipedema remains unclear and no specific molecular biomarkers have yet been established for its diagnosis, growing evidence suggests that microvascular dysfunction and chronic low-grade inflammation are key contributors to disease development and progression [3,4,5].

Increased capillary permeability, impaired lymphatic drainage, and accumulation of interstitial fluid are consistently reported features of the affected adipose tissue in patients with lipedema, suggesting dysregulation of angiogenic and lymphangiogenic signaling pathways [3,4,5]. Vascular endothelial growth factor A (VEGF-A), VEGF-C, and VEGF-D are major mediators involved in the formation and remodeling of blood and lymphatic vessels [5,6]. Therefore, they may represent candidate markers of the microangiopathy and fluid imbalance associated with lipedema [3,4,5,6]. Recently, platelet factor 4 (PF4) has been identified as a biomarker of lymphatic vascular dysfunction [7]. Elevated PF4 levels have been detected in circulating blood plasma-derived exosomes of patients with lipedema, further supporting the hypothesis that lymphatic dysfunction may contribute to disease pathology [7].

Lipedema is also recognized as a chronic adipose tissue disorder characterized by low-grade inflammation and immune cell infiltration within subcutaneous adipose tissue [3,6,8]. In most patients with stage 3 disease, elevated circulating inflammatory mediators such as tumor necrosis factor ligand superfamily member 14 (TNFSF14), caspase 8 (CASP8), EN-RAGE (S100A12), 4E-BP1, adenosine deaminase (ADA), monocyte chemoattractant protein-1 (MCP-1), as well as oxidative stress markers including malondialdehyde, superoxide dismutase, and catalase have been reported [8]. Another study demonstrated higher systemic levels of interleukin-11 (IL-11), interleukin-28A (IL-28A), and interleukin-29 (IL-29) in patients with lipedema [9]. Plasma interleukin-6 (IL-6) and C-reactive protein (CRP) levels were normal in some cohorts [6,10], although advanced disease stages of lipedema were associated with increased systemic CRP concentrations [11]. Thigh adipose tissue biopsies revealed significantly differentially expressed genes in stages II and III, including increased expression of inflammatory markers such as IL6 and tumor necrosis factor-alpha (TNFα), as well as macrophage markers CD86 and mannose receptor C-type 1 (MRC1; CD206) [8]. Histological assessment further identified elevated numbers of M2-polarized macrophages in lipedema subcutaneous adipose tissue, accompanied by stage-dependent adipocyte hypertrophy and progressive interstitial fibrosis [8]. Together, these findings support the concept of a distinct inflammatory microenvironment in lipedema.

In our previous studies, we demonstrated that a low-carbohydrate, high-fat (LCHF) diet was associated with significant reductions in BMI, lower-limb volume, and adipose-tissue pain, as well as improvements in body composition [12,13]. In addition, we observed favorable changes in selected metabolic and basic laboratory parameters compared with control groups [14]. However, these studies focused primarily on clinical and metabolic outcomes. The effects of long-term adherence to an LCHF dietary intervention on angiogenic, lymphangiogenic, inflammatory, and lipid signaling pathways in lipedema remain largely unknown.

The aim of the present study was to assess changes in selected vascular, lymphatic, inflammatory, and lipid mediators after seven months of a medically supervised LCHF dietary intervention in women with lipedema. By investigating the biochemical effects of this dietary intervention, we sought to provide new insights into the interplay between metabolism, circulating biomarkers, and clinical response in lipedema. To our knowledge, the long-term systemic effects of a medically supervised LCHF intervention on vascular, lymphatic, and immunometabolic pathways in lipedema have not previously been characterized.

## 2. Materials and Methods

### 2.1. Patients

Twenty-four women with lipedema who completed the 7-month LCHF dietary intervention were included in the analysis. Participants were recruited from the Angiology Outpatient Clinic.

The diagnosis of lipedema was based on the criteria of Wold et al. [1], with later modifications [15]. Minimal inclusion criteria were: disproportionate adipose tissue distribution in the lower extremities compared with the upper body, spontaneous pain or pain on pressure within the adipose tissue of the legs, and a negative Stemmer’s sign. Exclusion criteria were: pregnancy, breastfeeding, less than 6 months postpartum, lymphedema, edema due to chronic venous insufficiency or heart failure, diabetes mellitus, renal or hepatic failure, uncontrolled thyroid dysfunction, and malignancy.

The median age of participants was 39.0 years. Disease severity was classified into four clinical stages and five clinical types [2]. Nutritional status was additionally classified according to BMI categories defined by the World Health Organization (normal weight, overweight, obesity classes I–III). Basic clinical characteristics of the patients are presented in Table 1.

The study was conducted in accordance with the Declaration of Helsinki. Ethical approval was obtained from the Bioethics Committee of Wroclaw Medical University, Poland (approvals no. KB-690/2017 and KB-456/2019). Written informed consent was obtained from all participants. The study was registered at ClinicalTrials.gov (NCT07530341, 30 March 2026).

### 2.2. Measurement of Clinical Parameters

Body weight was assessed using a TANITA MC-780MA analyzer (Tanita, Tokyo, Japan), and height was measured with a TANITA HR-001 stadiometer (Tanita, Tokyo, Japan). Body Mass Index (BMI) was calculated as weight (kg) divided by height squared (m^2^).

Waist circumference was measured at the midpoint between the lowest rib and the iliac crest. Waist-to-height ratio (WHtR) was calculated as waist circumference divided by height.

Leg circumferences were measured with a standard tape measure to the nearest 0.5 cm. Leg volume was calculated from circumferences obtained every 4 cm using the formula for a truncated cone [16]. Leg volume was calculated for each lower limb, and the mean of the right and left leg volume was used for statistical analysis.

Assessment of the pain level of the adipose tissue in the legs was performed using the validated Visual Analogue Scale (VAS). The VAS is one of the most widely used instruments for pain assessment and consists of a 10 cm horizontal line anchored at 0 (“no pain”) and 10 (“worst imaginable pain”), on which participants indicate their perceived level of pain [17]. VAS measurements were performed at baseline and after completion of the dietary intervention.

### 2.3. Nutritional Intervention

The dietary intervention consisted of an individualized, calorie-restricted low-carbohydrate, high-fat (LCHF) dietary pattern, with daily carbohydrate intake limited to <50 g; however, it was specifically designed in a Mediterranean-style pattern emphasizing high-quality foods with potential anti-inflammatory properties. Daily energy intake was distributed across three meals, each including a source of protein, fat, and low-carbohydrate vegetables. Protein sources included eggs, dairy products, lean poultry, and lean beef, while foods high in saturated fat (e.g., processed meats, lard, poultry skin, and organ meats) were minimized. Fat intake was primarily derived from monounsaturated fatty acids (olive oil, avocado, nuts) and polyunsaturated fatty acids from plant oils (canola and flaxseed oils), seeds, nuts, and fatty fish (salmon, herring, mackerel, and sardines). Each meal included non-starchy vegetables, and small portions of berries were allowed daily. The diet also incorporated herbs, spices, and one daily serving of black or green tea.

Individualized 7-day meal plans, including menus, recipes, and shopping lists, were prepared by a clinical dietitian using the web-based DietetykPro software (https://dietetykpro.pl/, Wroclaw, Poland) and repeated cyclically throughout the 7-month intervention. The software was used between January 2021 and May 2022. Energy requirements were estimated based on resting metabolic rate (RMR) and physical activity level (PAL), and caloric intake was set at approximately 70–90% of calculated energy needs, depending on baseline body weight. DietetykPro was used solely as a dietary planning tool, and no sponsorship or external funding was involved in its use in this study.

Dietary adherence was monitored using monthly 24 h dietary recalls conducted by phone or during follow-up visits, with additional evaluations at 2.5 and 5 months. Adherence was evaluated qualitatively based on consistency with the prescribed dietary pattern, including maintenance of carbohydrate intake below 50 g/day. Strict 100% compliance with every component of the meal plan was not required; instead, adherence was assessed according to overall consistency with the prescribed LCHF pattern and the absence of repeated or substantial deviations. Common deviations included increased consumption of carbohydrate-rich foods, skipping meals, or unnecessary restriction of dietary fat. Repeated or substantial deviations from the dietary protocol were considered non-adherence. Participants who did not adhere to the dietary protocol were excluded from the study to ensure intervention consistency. The nutritional intervention has been described in detail elsewhere [13,14].

### 2.4. Blood Samples

Venous blood samples were collected after a 12 h overnight fast at baseline and after 7 months of the LCHF dietary intervention. Samples were stored in polypropylene tubes at −80 °C until analysis. All measurements were performed after completion of sample collection.

### 2.5. Chemicals

Standards of Thromboxane B2, Leukotriene B4, Prostaglandin D2, Prostaglandin E2, 6-keto Prostaglandin F1α, Prostaglandin F2α, 15-deoxy-Δ12,14-Prostaglandin J2, 13,14-dihydro Prostaglandin E1, and their isotope-labeled standards were procured from Cayman Chemical Company (Ann Arbor, MI, USA). Palmitoyl Ethanolamide (PEA), Palmitoyl Ethanolamide-d4 (PEA-d4), Oleoyl Ethanolamide (OEA), Oleoyl Ethanolamide-d2 (OEA-d2), 2-Arachidonoyl Glycerol (2-AG), 2-Arachidonoyl Glycerol-d5 (2-AG-d5), 1-Arachidonoyl Glycerol (1-AG), 1-Arachidonoyl Glycerol-d5 (1-AG-d5), Arachidonoyl Ethanolamide (AEA), Arachidonoyl Ethanolamide-d8 (AEA-d8), Stearoyl Ethanolamide (SEA), Stearoyl Ethanolamide-d3 (SEA-d3) were procured from Cayman Chemical Company (Ann Arbor, MI, USA). Toluene, methyl tert-butyl ether (MTBE), and formic acid (FA) were acquired from Merck Millipore (Warsaw, Poland). Methanol, acetonitrile (ACN), ethyl acetate, water, and formic acid (FA) were acquired from Witko (Warsaw, Poland).

### 2.6. Sample Preparation, LC-MS/MS Analysis, and Luminex Assays

#### 2.6.1. Targeted Metabolomic Analysis

Samples were subjected to quantitative analysis. Compounds were separated using a triple quadrupole mass spectrometer Xevo Absolute (Waters, Milford, MA, USA). Separation of eicosanoids was achieved based on the previously described method [18]. Briefly, 100 µL of samples or calibration standards, placed in 2 mL Eppendorf Safe-Lock tubes (Eppendorf, Hamburg, Germany), were mixed with 20 µL of 0.2% FA and 10 µL of internal standards in methanol for 1 min at 1100 RPM and 25 °C. Afterward, 200 µL of ACN and 250 µL of ethyl acetate were added to the samples and mixed for 10 min at 1100 RPM and 25 °C. The mixtures were centrifuged at 4 °C for 7 min at 15,000 RCF. A 370 µL aliquot of the obtained supernatant was evaporated to dryness and redissolved in 25 µL of 20% ACN in water before analysis. Endocannabinoids were prepared in accordance with a previously developed method [19]. A total of 100 µL of calibration standards or serum samples was transferred into 2.0 mL polypropylene tubes and combined with 20 µL of an internal standard solution in methanol (160 nM PEA-d4; 40 nM OEA-d2 and AEA-d8; and 400 nM 2AG-d5 and 1-AG-d5). After 1 min of mixing, the samples were extracted with 400 µL of toluene at 25 °C for 5 min and then centrifuged at 12,500 RPM for 5 min at 4 °C. Subsequently, 350 µL of the resulting supernatant was evaporated to dryness. The remaining residue was reconstituted in 120 µL of ACN/water/FA (1/4/0.1, *v*/*v*/*v*) and transferred to autosampler glass vials.

Chromatographic separation of metabolites was conducted on a BEH Shield C18 column (100 mm × 2.1 mm i.d., 1.7 µm; Waters, Milford, MA, USA). Data acquisition for all compounds was carried out using MassLynx 4.2 SCN 1050 Software (Waters, Milford, MA, USA) in multiple reaction monitoring mode (MRM).

#### 2.6.2. Cardiovascular and Inflammatory Luminex Assays 

Selected analytes were profiled in duplicate using a flow cytometry-based method with magnetic microspheres conjugated to monoclonal antibodies and analyzed on a Luminex Magpix Multiplexing System (Luminex Corporation, Austin, TX, USA), according to the manufacturer’s instructions, incorporating Luminex xMAP technology [20].

The assay used validated custom plexes allowing simultaneous measurement of VEGF-A, VEGF-C, VEGF-D, Angiopoietin-2, sVCAM-1, sICAM-1, P-selectin, TNFα, and interleukin (IL)-1β, IL-6, IL-8, IL-13, and IL-28α. Standard curves were drawn using 4- or 5-parameter logistic regression, and the data were analyzed using Belysa Immunoassay Curve Fitting Software version 1.2 (Merck KGaA, Darmstadt, Germany).

### 2.7. Statistical Analysis

Data preprocessing was performed in R 4.4.2 (packages: tidyverse 2.0.0, janitor 2.2.1). Classical comparisons of dependent samples were conducted using Statistica 13.3 (StatSoft, Tulsa, OK, USA; licensed to Wroclaw Medical University), while statistical modeling was performed in R 4.4.2. A statistical significance level of α = 0.05 was established.

Normality of continuous variables was assessed using the Shapiro–Wilk test. For time-based comparisons of quantitative variables in dependent samples, the Wilcoxon signed-rank test was applied. Descriptive statistics included median, minimum, maximum, and interquartile range (Q1–Q3).

Considering the sample size, a maximum of two degrees of freedom was assumed for multivariate modeling to prevent overfitting. Changes in three outcomes (BMI, leg volume loss, and adipose tissue pain) were modeled using four strategies: (i) association with baseline values of parameters, (ii) association with changes in analyzed parameters over time, and (iii–iv) both models additionally adjusted for baseline values of the outcome variable. The third and fourth strategies were based on the assumption that higher baseline values of the response variable would be associated with greater changes over time.

BMI changes were analyzed using linear models (see Figure S1). Leg volume reduction was analyzed using generalized linear models with a gamma distribution and log-link function, as this approach best matched the observed right-skewed distribution of the data (see Figure S2).

Pain improvement was analyzed as a continuous outcome. Pain loss was defined as the change in pain between follow-up and baseline (pain change = t1 − t0), with more negative values indicating greater symptom reduction. Associations between pain loss and circulating biomarkers were evaluated using separate Gaussian linear regression models. For each biomarker, two models were fitted: an unadjusted model including the biomarker only and an adjusted model additionally including baseline pain severity. Both baseline biomarker concentrations and their longitudinal changes (change = t1 − t0) were analyzed separately. Given the limited sample size (N = 24), multivariable biomarker models were not constructed and the analyses were considered exploratory. Regression coefficients (β) are presented on the original measurement scales to preserve biological interpretability. All *p*-values are nominal, and no correction for multiple testing was applied; therefore, these analyses should be interpreted as exploratory and hypothesis-generating.

## 3. Results

### 3.1. Changes in Clinical Characteristics

After the LCHF dietary intervention diet, we observed significant reductions in body mass index (BMI), waist-to-height ratio (WHtR), and leg volume (*p* < 0.001). Participants also reported a reduction in adipose tissue pain (*p* < 0.001). The distribution of leg volume change was right-skewed and showed substantial inter-individual variability (Figure S2); therefore, changes are presented as medians and interquartile ranges. The clinical parameters before and after the intervention are presented in Table 2.

### 3.2. Changes in Circulating Biomarkers

After the LCHF diet, circulating concentrations of VEGF-A, VEGF-D, IL-8, and angiopoietin-2 increased, whereas levels of endothelial adhesion molecules (sVCAM-1, sICAM-1, P-selectin), pro-inflammatory cytokines (TNF-α, IL-1β), and several endocannabinoid-related lipid mediators (AEA, OEA, PEA, 1-AG, and 2-AG) decreased. Angiopoietin-2 showed a particularly pronounced increase, with a median rise of 86.9% relative to baseline. The reductions in sVCAM-1, sICAM-1, P-selectin, OEA, PEA, and 2-AG were among the most marked changes, with post-intervention concentrations approaching very low circulating values. Other metabolites showed more moderate changes, with median values generally exceeding 35% of baseline. Among lipid mediators, AEA demonstrated the smallest relative change (36.9% decrease).

Detailed concentrations before and after the intervention and their corresponding statistical comparisons are presented in Table 3. Overall, the LCHF diet was associated with increases in selected angiogenic mediators and decreases in inflammatory and endothelial adhesion markers.

### 3.3. Regression Models of Clinical Outcomes

For consistency of interpretation, outcome variables were defined as loss (baseline minus follow-up), such that higher values indicated greater clinical improvement. After adjustment for baseline BMI, both baseline concentrations and longitudinal changes in selected lipid mediators were associated with BMI reduction (Table 4). Higher baseline OEA concentration was associated with greater BMI reduction (β = 0.039, *p* = 0.017). Longitudinal change in OEA was also associated with BMI reduction (β = −0.038, *p* = 0.020). Higher baseline AEA concentration was likewise associated with greater BMI reduction (β = 0.322, *p* = 0.033). The association between longitudinal changes in AEA and BMI reduction approached statistical significance (*p* = 0.059). In unadjusted analyses, changes in 6-keto-PGF1α were associated with BMI reduction; however, this relationship was attenuated after adjustment for baseline BMI.

In the adjusted model, change in IL-8 was nominally associated with leg volume reduction, with greater increases in IL-8 associated with smaller leg volume reduction (β = −0.04, *p* = 0.024) (Table 5).

Adipose tissue pain improvement was evaluated using exploratory separate Gaussian linear regression models (Table 6). In unadjusted analyses, greater decreases in P-selectin over time were associated with greater pain reduction (β = −0.0115, *p* = 0.015), whereas higher baseline P-selectin levels were associated with smaller subsequent improvement (β = 0.0114, *p* = 0.016). In addition, increases in IL-13 over time were associated with greater pain reduction (β = 0.0106, *p* = 0.016).

After adjustment for baseline pain severity, several associations remained evident. Greater increases in IL-13 continued to be associated with greater pain improvement (β = 0.0091, *p* = 0.029). Similarly, decreases in P-selectin over time remained associated with greater pain reduction (β = −0.0093, *p* = 0.050), while higher baseline P-selectin levels were associated with smaller improvement. In addition, greater decreases in VEGF-A over time were associated with greater pain reduction after adjustment for baseline pain (β = −0.0065, *p* = 0.050).

Overall, the exploratory regression analyses indicated associations between changes in selected lipid and inflammatory mediators and clinical improvement during the LCHF dietary intervention. OEA and AEA were associated with BMI reduction, whereas change in IL-8 showed a nominal association with leg volume reduction. Changes in P-selectin, IL-13, and VEGF-A were associated with pain improvement. Given the limited sample size and the exploratory nature of these analyses, no adjustment for multiple testing was applied, and the findings should therefore be interpreted as hypothesis-generating.

## 4. Discussion

This study provides preliminary insights into the vascular-lymphatic and immunometabolic changes accompanying clinical improvement during long-term calorie-restricted LCHF dietary intervention in women with lipedema. Importantly, because of the single-arm exploratory design, the findings should be interpreted as associations observed over time during the LCHF intervention rather than as evidence of causality or of specific mechanistic effects of the diet.

We observed a significant increase in circulating vascular factors, specifically VEGF-A, VEGF-D, and angiopoietin-2, while VEGF-C levels remained unchanged. VEGF-A and VEGF-D are key mediators of angiogenesis and lymphangiogenesis, respectively [21,22,23,24,25]. In the context of concomitant clinical improvement, these changes may be consistent with altered vascular and lymphatic signaling during the intervention. However, circulating factors alone do not allow us to determine whether they reflect adaptive remodeling, compensation, or other systemic processes. Accordingly, interpretations referring to improved tissue perfusion, microvascular function, endothelial plasticity, or lymphatic responsiveness remain hypothetical and were not directly examined in this study. Additionally, the elevated levels of angiopoietin-2 suggest that vascular signaling changed over the course of the LCHF intervention [26], but the biological meaning of this increase cannot be established on the basis of serum measurements alone. Overall, these findings suggest that clinical improvement was accompanied by changes in circulating markers related to vascular and lymphatic biology.

The absence of change in VEGF-C, in contrast to the increase in VEGF-D, is consistent with differential regulation of circulating lymphangiogenic markers during the LCHF dietary intervention. However, this interpretation should be made cautiously. Although VEGF-C and VEGF-D share overlapping functions [22,23,24,25], our data do not provide mechanistic evidence that one pathway predominated over the other, nor do they allow conclusions regarding the source, tissue specificity, or functional consequences of these circulating changes. Therefore, the observed pattern is better described as differential changes in circulating lymphangiogenic markers rather than proof of distinct pathway activation. The concurrent elevation of angiopoietin-2 may indicate additional changes in vascular-related signaling, although this likewise requires cautious interpretation.

Felmerer et al. reported altered vascular and lymphatic signaling and a pro-inflammatory adipose tissue environment in lipedema [6]. Our findings are compatible with the concept that circulating vascular and inflammatory mediators can change in parallel with clinical improvement in lipedema. However, we cannot conclude that these changes represent “normalization” of vascular homeostasis, because no healthy control group or tissue-level validation was included. Accordingly, terms such as “partial normalization” should be understood as descriptive and hypothesis-generating rather than definitive.

Moreover, while the rise in VEGF-D levels observed in our study may reflect structural remodelling secondary to adipose tissue loss, emerging evidence suggests that LCHF or ketogenic dietary approaches may also exert direct effects on lymphatic endothelial cell biology. This possibility is supported mainly by preclinical and limited clinical observations and should therefore be considered speculative in relation to our cohort. In preclinical models, ketone bodies have been shown to stimulate lymphatic vessel growth [27]. In a preclinical mouse model of tail lymphedema, which closely mimics features of human secondary lymphedema, ketogenic dietary intervention enhanced lymphatic vessel regeneration and function, reduced the infiltration of anti-lymphangiogenic immune cells, and led to a significant reduction in tissue oedema [27]. Clinical observations further support these findings. In patients with unilateral secondary lymphedema, adherence to a ketogenic diet was associated with measurable improvements in lymphatic function and reduction in limb oedema volume in a subset of individuals [28]. Nevertheless, extrapolation from secondary lymphedema models or other clinical populations to lipedema should be made with caution, and our data do not demonstrate direct lymphatic effects of the dietary intervention.

In our study, the LCHF diet was also associated with reductions in several circulating inflammatory and endothelial activation markers, evidenced by significant decreases in TNF-α, IL-1β, sVCAM-1, sICAM-1, and P-selectin. The reduction in circulating TNF-α and IL-1β is consistent with decreased systemic inflammatory activity [29,30], aligning with some previous findings on the possible anti-inflammatory effects of carbohydrate restriction and ketone body metabolism [31]. The marked reductions in soluble adhesion molecules (sVCAM-1, sICAM-1, and P-selectin) in our patients are consistent with reduced endothelial activation and leukocyte trafficking, which can be interpreted as improved vascular function and reduced endothelial inflammation during LCHF dietary intervention [32,33,34]. At the same time, because no control group was included, these changes cannot be attributed specifically to the LCHF diet itself and may also relate to weight loss, time-dependent variation, or other unmeasured factors.

Notably, the proinflammatory cytokine IL-8 increased following the LCHF diet in our study. Although IL-8 is traditionally classified as a pro-inflammatory chemokine, it also exerts potent pro-angiogenic effects and contributes to vascular remodeling [35,36]. This result does not support a simple uniformly anti-inflammatory interpretation of the biomarker profile. Rather, it underscores that the observed systemic response was biologically mixed and may have involved parallel inflammatory, reparative, or angiogenic processes that cannot be disentangled in this study. Therefore, this finding is difficult to interpret unambiguously and may reflect complex, context-dependent changes in inflammatory and angiogenic signaling rather than a purely beneficial or detrimental effect. Given this ambiguity, IL-8 should be regarded as one of the more difficult-to-interpret findings rather than as evidence of beneficial remodeling.

No significant changes were observed in IL-6, IL-13, or IL-28α, suggesting that the intervention was not associated with major alterations in selected Th2-related or interferon-mediated pathways. However, this interpretation should be considered with caution given the limited sample size and variability of cytokine responses [37,38,39,40]. IL-6 is a pleiotropic cytokine involved in inflammation, metabolism, and tissue stress responses; the absence of a significant change in IL-6 may therefore indicate either biological stability or insufficient power to detect modest effects [37,38]. IL-13, often linked to type 2 immune signaling and tissue remodeling, also remained unchanged at the group level, despite appearing in exploratory models of pain improvement [39]. IL-28α, a type III interferon, has less clearly defined relevance in lipedema, and its stability may simply indicate that this pathway was not prominently affected or that any effect was below the resolution of this small exploratory study.

From the perspective of lipid mediators, we documented a significant decline in several anti-inflammatory and endocannabinoid-related molecules, including AEA, OEA, PEA, 13,14-dihydro PGE1, 15-deoxy-Δ12,14-PGJ2, and both 1- and 2-arachidonoylglycerol (1-AG and 2-AG). These compounds typically exert immunomodulatory and homeostatic roles, particularly within inflamed adipose tissue [41,42]. At first glance, the decrease in several mediators with generally anti-inflammatory or homeostatic properties may appear counterintuitive. One possible explanation is that lower circulating concentrations reflect altered endocannabinoid-related signaling in the setting of changing metabolic and inflammatory status, although this remains tentative [43]. However, this interpretation remains tentative, because circulating concentrations do not directly capture tissue-specific production, receptor activation, or net biological effect. Therefore, our data support the presence of altered endocannabinoid-related and pro-resolving lipid signaling during the intervention, but not a definitive conclusion regarding whether these changes were beneficial, compensatory, or secondary to weight loss and altered nutrient intake. Notably, 15d-PGJ2, a potent PPAR-γ ligand, is involved in negative regulation of NF-κB and macrophage activity [44]. Its reduction may be compatible with reduced need for counter-regulatory signaling, but alternative explanations are also plausible. Conversely, we found no significant changes in several classical pro-inflammatory prostanoids and leukotrienes (PGE2, PGD2, PGF2α, TXB2, LTB4, and 6-keto-PGF1α). This stability may suggest that the LCHF diet primarily modulates resolution pathways and chronic inflammatory tone, rather than acute eicosanoid-mediated inflammation. Given the exploratory design and multiple comparisons, however, this pattern should be interpreted cautiously.

Our findings also identified exploratory associations between endocannabinoid-related lipid mediators and diet-induced body composition changes in women with lipedema. Baseline OEA levels and longitudinal decreases in OEA emerged as potential predictors of BMI reduction during the LCHF dietary intervention. Similarly, baseline AEA levels were also associated with BMI reduction over time. Because these analyses were exploratory and based on a small sample, these mediators should currently be viewed as candidate correlates of response rather than validated predictors. These findings are consistent with previous reports linking the endocannabinoid system to energy balance and adiposity regulation [41,42].

In exploratory modeling, change in IL-8 showed a nominal association with leg volume reduction, with greater increases in IL-8 associated with smaller reduction in leg volume. Given the small sample size, exploratory design, and biologically complex role of IL-8 [35,36], this finding should be interpreted with caution.

Beyond volumetric and metabolic improvements, reductions in adipose tissue pain were associated with changes in markers of endothelial activation and vascular-immune signaling. In exploratory analyses, greater pain improvement was linked to decreases in P-selectin and VEGF-A and to increases in IL-13, suggesting that symptom relief may be related to reduced endothelial activation and a shift toward a more anti-inflammatory tissue environment rather than to adipose tissue loss alone. Such interpretation is biologically plausible in light of previous work linking selectins, VEGF-A, and IL-13-related pathways to pain modulation [45,46,47]. These associations are intriguing, but they should not be interpreted as evidence that changes in these biomarkers mediated pain reduction. Rather, they indicate that symptom improvement and selected circulating markers changed in parallel within this cohort. Given the small sample size and exploratory design, these findings should be interpreted cautiously and considered hypothesis-generating.

Collectively, these observations do not establish a mechanistic model, but they do suggest that clinical improvement during the intervention coincided with changes in endothelial, inflammatory, angiogenic, and lipid-related circulating mediators. Any mechanistic interpretation, such as reduced endothelial activation, modulation of angiogenic signaling, altered immune-stromal interactions, improved microvascular stability, or changes in tissue fluid regulation, remains hypothetical and requires confirmation in controlled studies with functional and tissue-level endpoints. Although these associations were identified in exploratory analyses and require confirmation in larger cohorts, they suggest a biologically plausible association between metabolic intervention, vascular-lymphatic function, and nociceptive symptom burden in lipedema.

This study has several strengths. It is one of the few prospective interventional studies evaluating the clinical and biochemical effects of a long-term LCHF dietary intervention in women with lipedema. The individualized, medically supervised 7-month intervention enabled assessment of sustained changes, while the combined evaluation of clinical outcomes and a broad panel of circulating angiogenic, inflammatory, and lipid mediators provided an integrated characterization of systemic responses accompanying the intervention. The use of Luminex multiplex assays and LC-MS/MS further strengthened the analytical approach. These strengths should, however, be interpreted in the context of the exploratory design.

However, several limitations should be acknowledged. This study was designed as an exploratory, hypothesis-generating pilot investigation and no formal a priori sample size calculation was performed. The relatively small sample size and multiple exploratory comparisons limit statistical power and generalizability, and the observed associations should be interpreted with caution and confirmed in larger cohorts. Because multiple biomarkers were analyzed, the risk of false-positive findings should also be considered, particularly for secondary and exploratory associations. In addition, the absence of a control group (either women with lipedema not following a LCHF diet or individuals with obesity without lipedema) limits the ability to distinguish diet-specific effects from disease-related or time-dependent changes. Furthermore, all analyses were based on circulating biomarkers, which may not fully reflect local processes within adipose tissue; tissue-level investigations would provide more direct insight into local inflammatory, angiogenic, and lipid-signaling mechanisms. Finally, although the dietary intervention was medically supervised, adherence was not objectively verified using standardized dietary records or biochemical markers such as circulating ketone bodies, which may have introduced inter-individual variability in metabolic response. This point is particularly important when interpreting the findings as LCHF-specific or related to carbohydrate restriction, because the degree of carbohydrate restriction and ketosis achieved over time was not objectively documented. An additional limitation is that the intervention combined carbohydrate restriction with caloric restriction; therefore, the respective contributions of macronutrient composition and energy deficit could not be distinguished. Despite these limitations, the prospective design and integrated clinical and biomarker evaluation provide meaningful descriptive insight into the biological adaptations accompanying a long-term LCHF dietary intervention in lipedema.

Overall, the clinical improvement observed in this cohort was accompanied by changes in circulating metabolic, vascular-related, inflammatory, and lipid mediators. However, because the intervention combined LCHF dietary composition with a moderate energy deficit, the present study does not allow separation of the effects of macronutrient composition from those of caloric restriction, weight loss, or reduced adiposity. Caloric restriction itself is known to improve metabolic parameters and reduce systemic inflammation. Therefore, our findings should be interpreted as reflecting systemic changes accompanying a calorie-restricted LCHF intervention rather than effects attributable specifically to carbohydrate restriction or ketosis. Future controlled, ideally isocaloric, comparative studies are needed to distinguish between the effects of energy deficit and dietary composition in lipedema.

Moreover, future studies should validate our findings in larger cohorts and investigate whether baseline or dynamic profiles of angiogenic, inflammatory, and lipid mediators can be used to stratify patients, guide personalized nutritional strategies, and better understand the biological pathways associated with dietary intervention in lipedema. Such studies should ideally include appropriate control groups, objective adherence measures, and tissue- or function-based assessments to better define the biological relevance of circulating biomarker changes.

## 5. Conclusions

In this preliminary prospective study, a calorie-restricted LCHF dietary intervention in women with lipedema was associated with significant reductions in body weight, leg volume, and adipose tissue pain, accompanied by changes in circulating angiogenic, inflammatory, and lipid mediators. These findings suggest systemic responses to dietary intervention; however, they should not be interpreted as evidence of specific mechanistic pathways. Associations observed between lipid mediators and clinical outcomes, as well as between vascular or inflammatory markers and clinical improvement, should be considered exploratory. Further studies in larger, controlled cohorts are required to confirm these findings and to better understand the mechanisms underlying dietary effects in lipedema.

## Figures and Tables

**Table 1:** Basic clinical characteristics of women with lipedema enrolled in the study (*n* = 24).

Parameter	Value
Age [years], median (Q1–Q3)	39.0 (34.0–62.0)
Stage (severity of lipedema), *n* (%)	
Stage I	9 (37.5%)
Stage II	13 (54.2%)
Stage III	2 (8.3%)
Stage IV	0 (0%)
Type (distribution of lipedema), *n* (%)	
Type 1	0 (0%)
Type 2	6 (25.0%)
Type 3	17 (70.8%)
Type 4	8 (33.3%)
Type 5	1 (4.2%)
BMI category, *n* (%)	
Normal weight (<25 kg/m^2^)	5 (20.8%)
Overweight (25.0–29.9 kg/m^2^)	4 (16.7%)
Obesity class I (30.0–34.9 kg/m^2^)	9 (37.5%)
Obesity class II (35.0–39.9 kg/m^2^)	2 (8.3%)
Obesity class III (≥40 kg/m^2^)	4 (16.7%)

Notes: Stage classification: Stage I, smooth skin with thickened subcutaneous tissue; Stage II, uneven skin surface with nodular subcutaneous fat; Stage III, large lobular deformations with massive adipose tissue deposits; Stage IV, lipolymphedema. Type classification: Type 1, buttocks and hips; Type 2, buttocks to knees with folds around the knees; Type 3, whole legs (buttocks to ankles); Type 4, arms (sometimes including forearms); Type 5, calves. Percentages for lipedema types may exceed 100% due to overlapping patterns. BMI, body mass index.

**Table 2:** Clinical parameters before and after a 7-month LCHF intervention in women with lipedema (*n* = 24).

Parameter	Before Diet Median (Q1–Q3)	After Diet Median (Q1–Q3)	Change Median (Min–Max)	Change Median (Q1–Q3)	*p*-Value
BMI [kg/m^2^]	32.00 (28.80–34.90)	27.95 (22.66–31.45)	–3.85 (–9.80 to –0.80)	–3.85 (–6.20 to –2.55)	<0.001
WHtR	0.63 (0.54–0.65)	0.53 (0.44–0.57)	−0.07 (−0.17 to −0.02)	−0.07 (−0.11 to −0.05)	<0.001
Leg Volume [mL]	12,327.75 (10,135.00–14,568.00)	9810.00 (8598.00–12,270.00)	−1573.85 (−5427.67 to 187.66)	−1573.85 (−2787.00 to −894.18)	<0.001
Pain (VAS)	5.50 (3.50–8.00)	3.00 (1.00–5.50)	–2.00 (–8.00 to 1.00)	–2.00 (–3.00 to –0.50)	<0.001

Note: Data are expressed as medians with interquartile ranges (Q1–Q3). Change values are presented separately as median (min–max) and median (Q1–Q3). *p*-values were calculated using the Wilcoxon signed-rank test. BMI, body mass index; WHtR, waist-to-height ratio; VAS, visual analogue scale.

**Table 3:** Differences in circulating vascular, lymphatic, inflammatory, and lipid mediator parameters before and after a 7-month LCHF intervention in women with lipedema (*n* = 24).

Variable	Before Diet (Mean ± SE)	After Diet (Mean ± SE)	Change (Mean ± SE)	*p*-Value
VEGF-A [pg/mL]	132.40 ± 20.37	370.44 ± 21.57	+238.04 ± 26.03	<0.001
VEGF-C [pg/mL]	758.28 ± 59.53	765.08 ± 40.77	+6.80 ± 60.08	0.911
VEGF-D [pg/mL]	25.19 ± 3.68	91.80 ± 3.11	+66.61 ± 4.29	<0.001
Angiopoietin-2 [pg/mL]	557.79 ± 41.26	1042.39 ± 66.82	+484.60 ± 77.26	<0.001
sVCAM-1 [ng/mL]	915.37 ± 67.28	10.82 ± 0.82	−904.56 ± 67.41	<0.001
sICAM-1 [ng/mL]	133.58 ± 11.53	1.59 ± 0.11	−131.99 ± 11.49	<0.001
P-selectin [pg/mL]	169.82 ± 19.20	2.37 ± 0.10	−167.45 ± 19.15	<0.001
TNF-α [pg/mL]	23.11 ± 2.52	10.41 ± 0.94	−12.70 ± 2.34	<0.001
IL-1β [pg/mL]	1.46 ± 0.29	0.64 ± 0.24	−0.82 ± 0.28	0.007
IL-6 [pg/mL]	27.68 ± 15.44	9.57 ± 4.95	−18.10 ± 11.48	0.128
IL-8 [pg/mL]	6.82 ± 1.20	27.53 ± 1.41	+20.71 ± 1.76	<0.001
IL-13 [pg/mL]	65.75 ± 27.63	81.92 ± 30.16	+16.17 ± 20.75	0.443
IL-28α [pg/mL]	0.64 ± 0.36	0.57 ± 0.32	−0.07 ± 0.28	0.806
Arachidonoyl ethanolamide (AEA) [ng/mL]	9.10 ± 0.55	5.74 ± 0.82	−3.36 ± 1.04	0.004
Oleoyl ethanolamide (OEA) [ng/mL]	99.26 ± 5.15	4.89 ± 0.38	−94.37 ± 5.19	<0.001
Palmitoyl ethanolamide (PEA) [ng/mL]	19.23 ± 0.83	1.16 ± 0.10	−18.07 ± 0.84	<0.001
13,14-dihydro Prostaglandin E1 [ng/mL]	0.020 ± 0.002	0.012 ± 0.002	−0.008 ± 0.003	0.012
15-deoxy-Δ12,14-Prostaglandin J2 [ng/mL]	0.222 ± 0.023	0.134 ± 0.012	−0.088 ± 0.026	0.002
Prostaglandin D2 [ng/mL]	0.137 ± 0.031	0.094 ± 0.021	−0.043 ± 0.030	0.174
Prostaglandin E2 [ng/mL]	0.115 ± 0.029	0.109 ± 0.017	−0.006 ± 0.030	0.839
6-keto Prostaglandin F1α [ng/mL]	0.034 ± 0.005	0.053 ± 0.021	+0.019 ± 0.022	0.405
Prostaglandin F2α [ng/mL]	0.186 ± 0.058	0.127 ± 0.027	−0.059 ± 0.056	0.302
Leukotriene B4 [ng/mL]	0.747 ± 0.171	0.424 ± 0.122	−0.323 ± 0.189	0.102
Thromboxane B2 [ng/mL]	12.68 ± 2.95	7.66 ± 2.12	−5.02 ± 2.91	0.098
1-Arachidonoyl glycerol (1-AG) [ng/mL]	38.08 ± 5.89	11.48 ± 1.04	−26.59 ± 5.60	<0.001
2-Arachidonoyl glycerol (2-AG) [ng/mL]	47.94 ± 6.19	1.44 ± 0.06	−46.50 ± 6.18	<0.001

Note: Data are presented as means ± standard error (SE). *p*-values were calculated using the Wilcoxon signed-rank test. VEGF, vascular endothelial growth factor; ICAM, intercellular adhesion molecule; VCAM, vascular cell adhesion molecule; TNF-α, tumor necrosis factor alpha; IL, interleukin. Lipid mediators include prostaglandins, leukotrienes, thromboxane, endocannabinoids, and related metabolites.

**Table 4:** Modeling intervention-associated BMI reduction.

Model	Variable	β	SE	t	*p*
Unadjusted	6-keto-PGF1α (change)	12.24	4.28	2.86	0.009
Adjusted	OEA (baseline)	0.039	0.015	2.54	0.017
Adjusted	OEA (change)	−0.038	0.015	−2.52	0.020
Adjusted	AEA (baseline)	0.322	0.141	2.29	0.033

Note: Adjusted models include baseline BMI. Only biomarkers showing nominal associations (*p* < 0.05) in separate regression models are presented. Regression coefficients (β) are presented on the original measurement scales. *p*-values are nominal.

**Table 5:** Modeling intervention-associated leg volume reduction.

Model	Variable	β	SE	t	*p*
Adjusted	IL-8 (change)	−0.04	0.0150	−2.43	0.024

Note: Only biomarkers showing nominal associations (*p* < 0.05) in separate regression models are presented. The adjusted model included baseline leg volume. Regression coefficients (β) are presented on the original measurement scales. *p*-values are nominal.

**Table 6:** Modeling intervention-associated adipose tissue pain reduction.

Model	Variable	β	SE	t	*p*
Unadjusted	P-selectin (change)	−0.0115	0.0044	−2.63	0.015
Unadjusted	P-selectin (baseline)	0.0114	0.0044	2.62	0.016
Adjusted	P-selectin (change)	−0.0093	0.0045	−2.08	0.050
Unadjusted	IL-13 (change)	0.0106	0.0040	2.61	0.016
Adjusted	IL-13 (change)	0.0091	0.0039	2.34	0.029
Adjusted	VEGF-A (change)	−0.0065	0.0031	−2.08	0.050

Note: Pain reduction (pain loss) was defined as the negative of pain change (pain change = t1 − t0), such that higher values indicate greater improvement. Changes represent differences between follow-up and baseline. Unadjusted models included the biomarker only. Adjusted models additionally included baseline pain severity (baseline pain score). Regression coefficients (β) are presented on the original measurement scales. *p*-values are nominal and provided for descriptive purposes.

## Data Availability

The original contributions presented in this study are included in the article/Supplementary Materials. Further inquiries can be directed to the corresponding author.

## Supplementary Materials

The following supporting information can be downloaded at https://www.mdpi.com/article/10.3390/nu18091381/s1, Figure S1: Distribution of changes in BMI after the 7-month LCHF dietary intervention; Figure S2: Distribution of changes in leg volume after the 7-month LCHF dietary intervention.

## Abbreviations

The following abbreviations are used in this manuscript:

1-AG	1-Arachidonoyl glycerol
2-AG	2-Arachidonoyl glycerol
4E-BP1	Eukaryotic translation initiation factor 4E-binding protein 1
ADA	Adenosine deaminase
AEA	Arachidonoyl ethanolamide
Ang-2	Angiopoietin-2
BMI	Body mass index
CASP8	Caspase 8
CRP	C-reactive protein
ICAM	Intercellular adhesion molecule
IL	Interleukin
LCHF	low-carbohydrate, high-fat
LC-MS/MS	Liquid chromatography–tandem mass spectrometry
LTB4	Leukotriene B4
MCP-1	Monocyte chemoattractant protein-1
MRC1	Mannose receptor C-type 1
OEA	Oleoyl ethanolamide
PEA	Palmitoyl ethanolamide
PF4	Platelet factor 4
PGD2	Prostaglandin D2
PGE2	Prostaglandin E2
PPAR-γ	Peroxisome proliferator-activated receptor gamma
S100A12	S100 calcium-binding protein A12 (EN-RAGE)
SE	Standard error
sICAM-1	Soluble intercellular adhesion molecule-1
sVCAM-1	Soluble vascular cell adhesion molecule-1
TNF-α	Tumor necrosis factor alpha
TNFSF14	Tumor necrosis factor ligand superfamily member 14
TXB2	Thromboxane B2
VAS	Visual analogue scale
VCAM	Vascular cell adhesion molecule
VEGF	Vascular endothelial growth factor
VEGF-A	Vascular endothelial growth factor A
VEGF-C	Vascular endothelial growth factor C
VEGF-D	Vascular endothelial growth factor D
WHtR	Waist-to-height ratio

## References

[1] Wold L.E., Hines E.A., Allen E.V. (1951). Lipedema of the legs: A syndrome characterized by fat legs and edema. Ann. Intern. Med..

[2] Kruppa P., Georgiou I., Biermann N., Prantl L., Klein-Weigel P., Ghods M. (2020). Lipedema—Pathogenesis, diagnosis, and treatment options. Dtsch. Arztebl. Int..

[3] Al-Ghadban S., Cromer W., Allen M., Ussery C., Badowski M., Harris D., Herbst K.L. (2019). Dilated blood and lymphatic microvessels, angiogenesis, increased macrophages, and adipocyte hypertrophy in lipedema thigh skin and fat tissue. J. Obes..

[4] Allen M., Schwartz M., Herbst K.L. (2020). Interstitial fluid in lipedema and control skin. Womens Health Rep..

[5] Duhon B.H., Phan T.T., Taylor S.L., Crescenzi R.L., Rutkowski J.M. (2022). Current mechanistic understandings of lymphedema and lipedema: Tales of fluid, fat, and fibrosis. Int. J. Mol. Sci..

[6] Felmerer G., Stylianaki A., Hollmén M., Ströbel P., Stepniewski A., Wang A., Frueh F.S., Kim B.-S., Giovanoli P., Lindenblatt N. (2020). Increased levels of VEGF-C and macrophage infiltration in lipedema patients without changes in lymphatic vascular morphology. Sci. Rep..

[7] Ma W., Gil H.J., Escobedo N., Benito-Martín A., Ximénez-Embún P., Muñoz J., Peinado H., Rockson S.G., Oliver G. (2020). Platelet factor 4 is a biomarker for lymphatic-promoted disorders. JCI Insight.

[8] Kruppa P., Gohlke S., Łapiński K., Garcia-Carrizo F., Soultoukis G.A., Infanger M., Schulz T.J., Ghods M. (2023). Lipedema stage affects adipocyte hypertrophy, subcutaneous adipose tissue inflammation and interstitial fibrosis. Front. Immunol..

[9] Wolf S., Deuel J.W., Hollmén M., Felmerer G., Kim B.-S., Vasella M., Grünherz L., Giovanoli P., Lindenblatt N., Gousopoulos E. (2021). A distinct cytokine profile and stromal vascular fraction metabolic status without significant changes in the lipid composition characterizes lipedema. Int. J. Mol. Sci..

[10] Nankam P.A.N., Cornely M., Kloting N., Bluher M. (2022). Is subcutaneous adipose tissue expansion in people living with lipedema healthier and reflected by circulating parameters? Front. Endocrinol..

[11] Patton L., Ricolfi L., Bortolon M., Gabriele G., Zolesio P., Cione E., Cannataro R. (2024). Observational study on a large Italian population with lipedema: Biochemical and hormonal profile, anatomical and clinical evaluation, self-reported history. Int. J. Mol. Sci..

[12] Jeziorek M., Szuba A., Kujawa K., Regulska-Ilow B. (2022). The effect of a low-carbohydrate, high-fat diet versus moderate-carbohydrate and fat diet on body composition in patients with lipedema. Diabetes Metab. Syndr. Obes..

[13] Jeziorek M., Chachaj A., Sowicz M., Adaszyńska A., Truszyński A., Putek J., Kujawa K., Szuba A. (2023). The benefits of low-carbohydrate, high-fat (LCHF) diet on body composition, leg volume, and pain in women with lipedema. J. Obes..

[14] Jeziorek M., Szuba A., Sowicz M., Adaszynska A., Kujawa K., Chachaj A. (2023). The effect of a low-carbohydrate high-fat diet on laboratory parameters in women with lipedema in comparison to overweight/obese women. Nutrients.

[15] Keith L., Seo C., Wahi M.M., Huggins S., Carmody M., Faerber G., Forner-Cordero I., Michelini S., Rapprich S., Rockson S.G. (2024). Proposed framework for research case definitions of lipedema. Lymphat. Res. Biol..

[16] Casley-Smith J.R. (1994). Measuring and representing peripheral oedema and its alterations. Lymphology.

[17] Price D.D., McGrath P.A., Rafii A., Buckingham B. (1983). The validation of visual analogue scales as ratio scale measures for chronic and experimental pain. Pain.

[18] Krzystek-Korpacka M., Fleszar M.G., Fortuna P., Gostomska-Pampuch K., Lewandowski Ł., Piasecki T., Kosyk B., Szeląg A., Trocha M. (2021). Modulation of prostanoids profile and counter-regulation of SDF-1α/CXCR4 and VIP/VPAC2 expression by sitagliptin in non-diabetic rat model of hepatic ischemia-reperfusion injury. Int. J. Mol. Sci..

[19] Kościelniak-Merak B., Batko I., Fleszar M., Kocot-Kępska M., Gamian A., Kobylarz K., Sztefko K., Tomasik P.J. (2020). Effect of intravenous perioperative-administered lidocaine on serum levels of endocannabinoids and related N-acylethanolamines in children. Minerva Anestesiol..

[20] Elshal M.F., McCoy J.P. (2006). Multiplex bead array assays: Performance evaluation and comparison of sensitivity to ELISA. Methods.

[21] Huggenberger R., Siddiqui S.S., Brander D., Ullmann S., Zimmermann K., Antsiferova M., Werner S., Alitalo K., Detmar M. (2011). An important role of lymphatic vessel activation in limiting acute inflammation. Blood.

[22] Davydova N., Harris N.C., Roufail S., Paquet-Fifield S., Ishaq M., Streltsov V.A., Williams S.P., Karnezis T., Stacker S.A., Achen M.G. (2016). Differential receptor binding and regulatory mechanisms for the lymphangiogenic growth factors vascular endothelial growth factor (VEGF)-C and -D. J. Biol. Chem..

[23] Rissanen T.T., Markkanen J.E., Gruchala M., Heikura T., Puranen A., Kettunen M.I., Kholová I., Kauppinen R.A., Achen M.G., Stacker S.A. (2003). VEGF-D is the strongest angiogenic and lymphangiogenic effector among VEGFs delivered into skeletal muscle via adenoviruses. Circ. Res..

[24] Karkkainen M.J., Haiko P., Sainio K., Partanen J., Taipale J., Petrova T.V., Jeltsch M., Jackson D.G., Talikka M., Rauvala H. (2004). Vascular endothelial growth factor C is required for sprouting of the first lymphatic vessels from embryonic veins. Nat. Immunol..

[25] Bokhari S.M.Z., Hamar P. (2023). Vascular endothelial growth factor-D (VEGF-D): An angiogenesis bypass in malignant tumors. Int. J. Mol. Sci..

[26] Nguyen V.P., Chen S.H., Trinh J., Kim H., Coomber B.L., Dumont D.J. (2007). Differential response of lymphatic, venous and arterial endothelial cells to angiopoietin-1 and angiopoietin-2. BMC Cell Biol..

[27] García-Caballero M., Zecchin A., Souffreau J., Truong A.-C.K., Teuwen L.-A., Vermaelen W., Martín-Pérez R., de Zeeuw P., Bouché A., Vinckier S. (2019). Role and therapeutic potential of dietary ketone bodies in lymph vessel growth. Nat. Metab..

[28] Lodewijckx I., Matthys C., Verheijen J., Verscuren R., Devoogdt N., Van der Schueren B., Goffin K., Fourneau I., Thomis S. (2024). Potential therapeutic effect of a ketogenic diet for the treatment of lymphoedema: Results of an exploratory study. J. Hum. Nutr. Diet..

[29] van Loo G., Bertrand M.J.M. (2023). Death by TNF: A road to inflammation. Nat. Rev. Immunol..

[30] Bester J., Pretorius E. (2016). Effects of IL-1β, IL-6 and IL-8 on erythrocytes, platelets and clot viscoelasticity. Sci. Rep..

[31] Youm Y.-H., Nguyen K.Y., Grant R.W., Goldberg E.L., Bodogai M., Kim D., D’Agostino D., Planavsky N., Lupfer C., Kanneganti T.-D. (2015). The ketone metabolite β-hydroxybutyrate blocks NLRP3 inflammasome-mediated inflammatory disease. Nat. Med..

[32] Wopereis S., Wolvers D., van Erk M., Gribnau M., Kremer B., A van Dorsten F., Boelsma E., Garczarek U., Cnubben N., Frenken L. (2013). Assessment of inflammatory resilience in healthy subjects using dietary lipid and glucose challenges. BMC Med. Genom..

[33] Troncoso M.F., Ortiz-Quintero J., Garrido-Moreno V., Sanhueza-Olivares F., Guerrero-Moncayo A., Chiong M., Castro P.F., García L., Gabrielli L., Corbalán R. (2021). VCAM-1 as a predictor biomarker in cardiovascular disease. Biochim. Biophys. Acta Mol. Basis Dis..

[34] Perkins L.A., Anderson C.J., Novelli E.M. (2019). Targeting P-selectin adhesion molecule in molecular imaging: P-selectin expression as a valuable imaging biomarker of inflammation in cardiovascular disease. J. Nucl. Med..

[35] Koch A.E., Polverini P.J., Kunkel S.L., Harlow L.A., DiPietro L.A., Elner V.M., Elner S.G., Strieter R.M. (1992). Interleukin-8 as a macrophage-derived mediator of angiogenesis. Science.

[36] Matsushima K., Yang D., Oppenheim J.J. (2022). Interleukin-8: An evolving chemokine. Cytokine.

[37] Scheller J., Chalaris A., Schmidt-Arras D., Rose-John S. (2011). The pro- and anti-inflammatory properties of the cytokine interleukin-6. Biochim. Biophys. Acta.

[38] Trujillo M.E., Sullivan S., Harten I., Schneider S.H., Greenberg A.S., Fried S.K. (2004). Interleukin-6 regulates human adipose tissue lipid metabolism and leptin production in vitro. J. Clin. Endocrinol. Metab..

[39] Lee C.G., Homer R.J., Zhu Z., Lanone S., Wang X., Koteliansky V., Shipley J.M., Gotwals P., Noble P., Chen Q. (2001). Interleukin-13 induces tissue fibrosis by selectively stimulating and activating transforming growth factor β1. J. Exp. Med..

[40] Souza-Fonseca-Guimaraes F., Young A., Mittal D., Martinet L., Bruedigam C., Takeda K., Andoniou C.E., Degli-Esposti M.A., Hill G.R., Smyth M.J. (2015). NK cells require IL-28R for optimal in vivo activity. Proc. Natl. Acad. Sci. USA.

[41] Serefko A., Lachowicz-Radulska J., Jach M.E., Swiader K., Szopa A. (2025). The endocannabinoid system in the development and treatment of obesity: Searching for new ideas. Int. J. Mol. Sci..

[42] Schulz P., Hryhorowicz S., Rychter A.M., Zawada A., Słomski R., Dobrowolska A., Krela-Kaźmierczak I. (2021). What role does the endocannabinoid system play in the pathogenesis of obesity?. Nutrients.

[43] Fisk H.L., Childs C.E., Miles E.A., Ayres R., Noakes P.S., Paras-Chavez C., Kuda O., Kopecký J., Antoun E., Lillycrop K.A. (2021). Dysregulation of endocannabinoid concentrations in human subcutaneous adipose tissue in obesity and modulation by omega-3 polyunsaturated fatty acids. Clin. Sci..

[44] Scher J.U., Pillinger M.H. (2005). 15d-PGJ2: The anti-inflammatory prostaglandin?. Clin. Immunol..

[45] Machelska H., Brack A., A Mousa S., Schopohl J.K., Rittner H.L., Schäfer M., Stein C. (2004). Selectins and integrins but not platelet-endothelial cell adhesion molecule-1 regulate opioid inhibition of inflammatory pain. Br. J. Pharmacol..

[46] Llorian-Salvador M., Gonzalez-Rodriguez S. (2018). Painful understanding of VEGF. Front. Pharmacol..

[47] Singh S.K., Krukowski K., Laumet G.O., Weis D., Alexander J.F., Heijnen C.J., Kavelaars A. (2022). CD8+ T cell-derived IL-13 increases macrophage IL-10 to resolve neuropathic pain. JCI Insight.

