# Supplementary material for: Changes in Vascular, Lymphatic, Inflammatory, and Lipid Mediators During a 7-Month Calorie-Restricted Low-Carbohydrate, High-Fat Dietary Intervention in Women with Lipedema: A Preliminary Prospective Study
Source: Nutrients. 2026 Apr 28;18(9):1381. doi: 10.3390/nu18091381 (PMC13164597; doi:10.3390/nu18091381)
Supplement: Supplementary file 1 [file nutrients-18-01381-s001.zip › nutrients-4231040-supplementary.pdf]

SUPPLEMENTARY MATERIALS

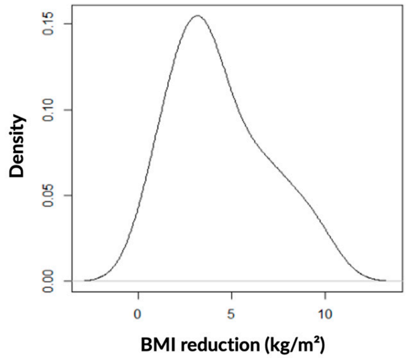

Figure S1. Distribution of changes in BMI after the 7-month LCHF dietary intervention.
Kernel density plot of individual BMI reduction (kg/m^2) in the study cohort (n = 24). Higher values indicate greater BMI reduction.

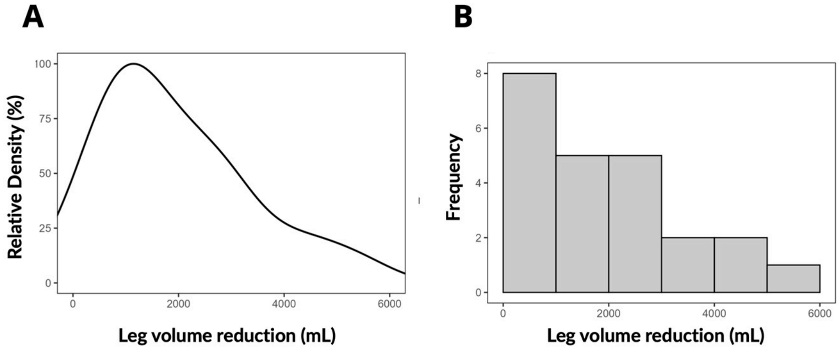

Figure S2. Distribution of changes in leg volume after the 7-month LCHF dietary intervention.
(A) Kernel density plot and (B) histogram of individual leg volume reduction (mL) in the study cohort (n = 24). Higher values indicate greater volume reduction.
